# Supplementary material for: Epistemically unwarranted beliefs scale, development and evidence of validity in the Chilean population
Source: PLoS One. 2025 Oct 24;20(10):e0333911. doi: 10.1371/journal.pone.0333911 (PMC12551922; doi:10.1371/journal.pone.0333911)
Supplement: S1 Appendix — (DOCX) [file pone.0333911.s001.docx]

**S1. Item Reduction Process.**

The following criteria were considered in the item reduction process:

1. Items referencing culturally specific events.
2. Items potentially limited by age relevance.
3. Items involving religious beliefs.
4. Items expressing hate ideology.
5. Reverse-coded items
6. Items with low variability or low homogeneity indices

The justification for the elimination of items according to the aforementioned criteria is broken down below.

*Table A1.*

*Justification for the elimination of items*

| **n** | **37 items proposed by the Lobato et al. scale (2)** | **Expert judges** | **Pilot study** |
| --- | --- | --- | --- |
| 1 | The variety of species of life that exist today is best explained by the scientific theory of evolution. | 5 |  |
| 2 | The earth is experiencing a period of global warming that is caused by many factors including human activities. | 5 |  |
| 3 | Childhood vaccines are one causal factor in the development of autism. |  | 6 |
| 4 | Human immunodeficiency virus (HIV) later develops into acquired immunodeficiency syndrome (AIDS). | 5 |  |
| 5 | A person chooses to be homosexual, bisexual, or heterosexual. | 4 |  |
| 6 | US astronauts have been to the moon. * |  |  |
| 7 | Members of the US government were involved in the planning and execution of the events that happened on 11 September 2001. * |  |  |
| 8 | President John F. Kennedy was assassinated by Lee Harvey Oswald, who acted alone. | 1 |  |
| 9 | Members of the US government were involved in a conspiracy to assassinate Martin Luther King, Jr. | 1 |  |
| 10 | Creatures known popularly as Big Foot, the Loch Ness Monster, and/or the Chupacabra exist. |  | 6 |
| 11 | Extraterrestrial life forms have visited Earth and abducted human beings. * |  |  |
| 12 | The age of the earth is approximately 4.3 billion years. | 5 |  |
| 13 | The shape of the earth is roughly a sphere. | 5 |  |
| 14 | After people die, they still interact with the living as ghosts. * |  |  |
| 15 | Certain objects, such as rabbits' feet and four-leafed clovers, genuinely bring good luck. |  | 6 |
| 16 | Homeopathic treatments are just as valid as traditional medical treatments for serious illnesses. * |  |  |
| 17 | Some humans have special mental abilities, such as the ability to move objects using only mental force or to predict the future with perfect accuracy. * |  |  |
| 18 | Prayer is effective at treating people with terminal diseases. | 3 |  |
| 19 | Reiki healing, otherwise known as Palm healing, is effective in healing the body. * |  |  |
| 20 | The Holocaust of the 1930s and 1940s was responsible for the deaths of over 11 million people, of whom six million were Jewish. | 4 |  |
| 21 | President Barack H. Obama is a native-born US citizen. | 1 |  |
| 22 | Most human beings only use approximately 10% of their brain. * |  |  |
| 23 | The beginning of the universe is best explained by the Big Bang Theory. | 5 |  |
| 24 | Human beings have souls that continue to exist after the body dies. | 3 |  |
| 25 | A full moon causes people to behave oddly. |  | 6 |
| 26 | Astrology is a valid explanation for the behaviors and personality of people. * |  |  |
| 27 | The Bermuda Triangle is a special location on the planet that, for some reason, causes ships and aircraft to crash or disappear more often than anywhere else on the planet. |  | 6 |
| 28 | Deceased individuals communicate with living people through séances or Oijua boards. |  | 6 |
| 29 | Black cats are unlucky. |  | 6 |
| 30 | The musician Elvis Presley is dead. | 2 |  |
| 31 | The singer John Lennon was the target of an assassination conspiracy. | 1 |  |
| 32 | The rappers Tupac Shakur and Biggie Smalls were victims of assassination conspiracies, which were covered up. | 2 |  |
| 33 | The singer Kurt Cobain was murdered by his wife, singer Courtney Love. | 2 |  |
| 34 | Lost city of Atlantis is a myth or a legend. |  | 6 |
| 35 | Some numbers and dates are more lucky or unlucky than others, such as Friday the 13th or the number 7. |  | 6 |
| 36 | Condoms used properly during sexual intercourse are very effective at preventing the spread of sexual transmitted diseases and pregnancy. | 5 |  |
| 37 | Geological objects, such as certain crystals, precious metals, or magnets, have intrinsic mystical properties. |  | 6 |
| Note: * Items from the final version applied | | | |
