# Supplementary material for: Epistemically unwarranted beliefs scale, development and evidence of validity in the Chilean population
Source: PLoS One. 2025 Oct 24;20(10):e0333911. doi: 10.1371/journal.pone.0333911 (PMC12551922; doi:10.1371/journal.pone.0333911)
Supplement: S2 Appendix — (DOCX) [file pone.0333911.s002.docx]

**S2. Multiple Post Hoc Comparisons.**

*Table B1.*

*Multiple post hoc comparisons*

|  | | *Mean difference* | *SE* | *df* | *t* | *p Tukey* | *Cohen’s D* |
| --- | --- | --- | --- | --- | --- | --- | --- |
| *Pseudoscientific beliefs* | | | | | | | |
| Gender | Female-Male | -0.24 | 0.11 | 533 | 2.20 | 0.028 | 0.291 |
| Political orientation | Left-Center | -0.10 | 0.23 | 533 | -0.43 | 0.974 | -0.121 |
|  | Left-Right | 0.08 | 0.17 | 533 | 0.46 | 0.968 | 0.092 |
|  | Left-Apolitical | -0.20 | 0.10 | 533 | -0.03 | 0.177 | -0.244 |
|  | Center-Right | 0.18 | 0.27 | 533 | 0.66 | 0.911 | 0.213 |
|  | Center-Apolitical | -0.10 | 0.24 | 533 | -0.44 | 0.972 | -0.123 |
|  | Right-Apolitical | -0.28 | 0.17 | 533 | -1.63 | 0.362 | -0.336 |
| Religious orientation | Adherence to a religious belief | 0.49 | 0.10 | 533 | 4.92 | <0.001 | 0.591 |
|  | Non-adherence to a religious belief | 0.07 | 0.19 | 533 | 0.35 | 0.934 | 0.080 |
|  | Non-belief in the divine | -0.43 | 0.20 | 533 | -2.13 | 0.085 | -0.511 |
| *Paranormal beliefs* | | | | | | | |
| Gender | Female-Male | 0.42 | 0.12 | 535 | 3.55 | <0.001 | 0.468 |
| Political orientation | Left-Center | -0.40 | 0.25 | 535 | -1.56 | 0.404 | -0.438 |
|  | Left-Right | -0.05 | 0.18 | 535 | -0.26 | 0.994 | -0.052 |
|  | Left-Apolitical | -0.32 | 0.11 | 535 | -2.92 | 0.019 | -0.348 |
|  | Center-Right | 0.35 | 0.30 | 535 | 1.21 | 0.624 | 0.386 |
|  | Center-Apolitical | 0.08 | 0.26 | 535 | 0.32 | 0.989 | 0.089 |
|  | Right-Apolitical | -0.27 | 0.19 | 535 | -1.44 | 0.474 | -0.297 |
| Religious orientation | Adherence to a religious belief | 0.54 | 0.11 | 535 | 4.96 | <0.001 | 0.596 |
|  | Non-adherence to a religious belief | 0.10 | 0.21 | 535 | 0.47 | 0.873 | 0.113 |
|  | Non-belief in the divine | -0.44 | 0.22 | 535 | -2.01 | 0.110 | -0.483 |
| *Conspiracy beliefs* | | | | | | | |
| Gender | Female-Male | 0.29 | 0.12 | 534 | 2.48 | 0.013 | 0.328 |
| Political orientation | Left-Center | -0.24 | 0.25 | 534 | -0.99 | 0.757 | -0.278 |
|  | Left-Right | 0.08 | 0.18 | 534 | 0.47 | 0.966 | 0.094 |
|  | Left-Apolitical | 0.00 | 0.11 | 534 | -0.01 | 1.000 | -0.001 |
|  | Center-Right | 0.33 | 0.28 | 534 | 1.16 | 0.653 | 0.372 |
|  | Center-Apolitical | 0.24 | 0.25 | 534 | 0.98 | 0.762 | 0.277 |
|  | Right-Apolitical | -0.08 | 0.18 | 534 | -0.46 | 0.967 | -0.095 |
| Religious orientation | Adherence to a religious belief | 0.33 | 0.11 | 534 | 3.16 | 0.005 | 0.380 |
|  | Non-adherence to a religious belief | 0.03 | 0.20 | 534 | 0.14 | 0.989 | 0.032 |
|  | Non-belief in the divine | -0.31 | 0.21 | 534 | -1.45 | 0.314 | -0.349 |
| Note: SE = Standard Error; df = Degrees of Freedom; t = t-statistic | | | | | | | |
