# Supplementary material for: Epistemically unwarranted beliefs scale, development and evidence of validity in the Chilean population
Source: PLoS One. 2025 Oct 24;20(10):e0333911. doi: 10.1371/journal.pone.0333911 (PMC12551922; doi:10.1371/journal.pone.0333911)
Supplement: S3 Appendix — (DOCX) [file pone.0333911.s003.docx]

**S3. Epistemically Unwarranted Beliefs Scale.**

The following statements refer to how you know and understand the world in general terms. Please indicate the level of agreement with which each statement applies to you.

*Table C1.*

*Epistemically unwarranted beliefs scale*

|  | Strongly Disagree | Disagree | Neither Agree nor Disagree | Agree | Strongly Agree |
| --- | --- | --- | --- | --- | --- |
| Pseudoscientific beliefs |  |  |  |  |  |
| 1. La homeopatía es tan válida como la medicina tradicional para tratar enfermedades graves  *(Homeopathy is as valid as traditional medicine for treating serious illnesses*). |  |  |  |  |  |
| 2. La mayoría de los seres humanos sólo usa aproximadamente el 10% de su cerebro  *(Most humans only use about 10% of their brain)*. |  |  |  |  |  |
| 3. Muchas enfermedades del cuerpo pueden ser tratadas con la aplicación adecuada de imanes especiales  *(Many diseases of the body can be treated with the proper application of special magnets)*. |  |  |  |  |  |
| Conspiracy beliefs |  |  |  |  |  |
| 1. Existen grupos secretos de personas poderosas que toman las decisiones más importantes sobre cómo se maneja el mundo a nivel internacional  (There are secret groups of powerful people who make the most important decisions about how the world is run on an international level). |  |  |  |  |  |
| 2. Existen tratamientos que son eficaces para tratar el cáncer pero que son ocultados por fines económicos  *(There are treatments that are effective in treating cancer but are hidden for economic purposes)*. |  |  |  |  |  |
| 3. Hay agentes del gobierno de los Estados Unidos que estuvieron involucrados en el ataque a las torres gemelas  *(There are agents of the U.S. government who were involved in the attack on the twin towers)*. |  |  |  |  |  |
| Paranormal beliefs |  |  |  |  |  |
| 1. La astrología entrega información valiosa respecto a cómo son las personas  *(Astrology provides valuable information about what people are like)*. |  |  |  |  |  |
| 2. Hay personas que tienen habilidades mentales especiales como predecir eventos con exactitude  *(There are people who have special mental abilities such as accurately predicting events)*. |  |  |  |  |  |
| 3. Hay formas inteligentes de vida extraterrestre que han visitado la Tierra  *(There are intelligent extraterrestrial life forms that have visited Earth)*. |  |  |  |  |  |
