# Supplementary material for: Epistemically unwarranted beliefs scale, development and evidence of validity in the Chilean population
Source: PLoS One. 2025 Oct 24;20(10):e0333911. doi: 10.1371/journal.pone.0333911 (PMC12551922; doi:10.1371/journal.pone.0333911)
Supplement: Attachment — Submitted filename: Dear Academic Editor and Reviewers.docx [file pone.0333911.s005.docx]

Dear Academic Editor and Reviewers,

We sincerely thank you for the insightful comments and suggestions provided during the evaluation of our manuscript. We truly appreciate the time and effort you devoted to reviewing our work. We have carefully considered each observation and present below a point-by-point response. We believe that the revisions have substantially improved the quality and clarity of the manuscript.

**Editor:**

**Comment 1:**
*The items removed from the full instrument should be included in the manuscript as the reviewer indicates.*

**Response:**
We added the removed items according to both reviewers' suggestions. For Reviewer 1, they were included in lines 146–149, 139–142, and 461–473; for Reviewer 2, in lines 461–473.

**Comment 2:**
*In the Methods section, please specify the type of study or methodology used (e.g., cross-sectional, descriptive, etc.) as well as the sampling method.*

**Response:**
This information was added (lines 162–163).

**Comment 3:**

One reviewer suggests using a scree plot to convey the results of the exploratory factor analysis.

**Response:**

We did not include a scree plot because we conducted an Exploratory Structural Equation Modeling (ESEM) instead of a traditional exploratory factor analysis. However, we are open to including it if deemed necessary.

**Comment 4:**
*In the Discussion section, a more detailed explanation about the sociodemographic variables would be a good addition (see the reviewer’s comment).*

**Response:**
We incorporated a comparative analysis with prior studies in the Discussion section (lines 305–312).

**Comment 5:**
*I also encourage to follow the reviewer’s advice to discuss alternative interpretations of the CRT, which I find particularly insightful in the context of this study.*

**Response:**
New information based on the studies suggested by Reviewer 1 was added (lines 301–304).

**Comment 6:**
*Although the manuscript reads quite smoothly overall, a careful review of the writing is advisable to ensure clarity and ease of understanding. I have noticed that the methodological and results sections are a bit harder to follow.*

**Response:**

The manuscript was revised for improved clarity and readability.

**Reviewer #1:**

**Comment 1:**
*While the justification for developing a culturally adapted scale is strong, the manuscript would benefit from deeper engagement with cross-cultural measurement theory. Specifically, consider expanding the discussion of how sociodemographic variables might affect EUB manifestations and their measurement. I suggest to consult previous research, such as the study by Torres et al. (2023; https://www.nature.com/articles/s41599-023-01681-3), where a series of cognitive and sociodemographic correlates of the different unwarranted beliefs (mainly pseudoscientific ones) are addressed. So an appropriate hypothesis regarding sociodemographic variables could be formulated and your results could be compared with previous research conducted with other population.*

**Response:**
We followed your suggestion by conducting mixed ANOVA analyses. A new theoretical section was added on sociodemographic variables related to EUB (gender, political and religious orientation) (lines 53–61 and 112–113). We also detailed the analyses in the Methods (195–200), presented results (261–274), added a figure (Figure 3), and included a supplementary table (Appendix 2). Comparisons with previous findings were discussed (lines 305–312).

**Comment 2:**
*The sample is heavily composed of university students (93.9%), which may limit generalizability. This limitation is acknowledged in the discussion, but I encourage the authors to explicitly mention this in the abstract as well. Additionally, considering the rich sociodemographic data collected and linking to the previous consideration, a more detailed analysis of associations between EUB and variables such as political orientation or religious belief could have enriched the findings.*

**Response:**
The abstract now reflects the student sample (lines 21–22) and the sociodemographic analyses (lines 26–27). The detailed analysis is described in the response to Comment 1.

**Comment 3:**
*Line 87: I recommend to reword this statement since it is not fully clear the direction of the correlation between variables. Is it positive (higher conspiracy beliefs, higher effectiveness of COVID-19 vaccines) or negative (higher conspiracy beliefs, lower effectiveness of the COVID-19 vaccines)?*

**Response:**
The statement was revised for clarity (lines 100–101).

**Comment 4:**
*The rationale behind the inclusion or exclusion of specific items could be improved. A brief justification for the new items created by the authors would strengthen the transparency of the development process:
• Line 125: I recommend to include an example of the mentioned rewording.
• Lines 127-132: a brief explanation of on what basis were these items created is desirable.*

**Response:**
Justifications were included (lines 146–149), a supplementary table for exclusions was added (lines 461–473), and a rewording example was included (lines 139–142).

**Comment 5:**
*Interpretation of the Cognitive Reflection Test (CRT): While CRT is an appropriate external correlate, their interpretation should be revised. Recent work (e.g., Bago & De Neys, 2019; Šrol & De Neys, 2021) suggests that CRT performance may arise through intuitive processes rather than through deliberate analytical reasoning. This challenges the traditional interpretation of CRT performance as primarily reflecting the ability to override intuitive but incorrect responses via deliberation. Instead, these studies point to an alternative account: CRT scores may reflect an individual’s sensitivity to conflict between heuristic intuitions and logical intuitions. Also, check the statement on lines 264 and 265 taking into account Bago and De Neys (2019) and Šrol and De Neys (2021) studies.*
[*https://www.tandfonline.com/doi/full/10.1080/13546783.2018.1507949*](https://www.tandfonline.com/doi/full/10.1080/13546783.2018.1507949)
[*https://www.tandfonline.com/doi/full/10.1080/13546783.2019.1708793*](https://www.tandfonline.com/doi/full/10.1080/13546783.2019.1708793)

**Response:**
We incorporated recent theoretical perspectives to encourage cautious interpretation (lines 301–304).

**Comment 6:**
*While the manuscript is readable, there are some typo errors that must be addressed:
- Line 35: “communication medias” should be “communication media”.
- Line 179: “model restrictyions” should be “model restrictions”.
- Line 2019: “pseudoscience belief” shoud be “pseudoscientific beliefs” or “beliefs in pseudoscience”.
A thorough language review would improve clarity and professionalism.*

**Response:**

All issues were addressed: line 35 (corrected in 39), line 179 (removed), line 201 (corrected in 236).

**Comment 8:**
*Check the reference in line 81, as it should be 38 instead of 20. Likewise, check that the reference of the line 82 is correct.*

**Response:**

References were corrected (lines 94–95).

**Comment 9:**
*Please ensure consistent and correct use of decimal points: e.g., use “0.833” rather than “0,833” (lines 218-220).*

**Response:**

This was corrected (lines 235–237).

**Reviewer #2:**

**Comment 1:**
 *It would also be useful to include the final version of the instrument with instructions for use. Regarding the item reduction process, greater transparency is suggested, including an appendix documenting the items that were eliminated.*

**Response:**
The final instrument and instructions were added in S3 Appendix (lines 477–482), and item reduction was documented in S1 Appendix (lines 461–473).

We hope that our revisions address all your concerns. We are grateful for your constructive input, which helped us strengthen the manuscript. Please do not hesitate to let us know if further clarification is needed.

Sincerely,

Karina Alarcón Castillo
On behalf of all co-authors
Universidad de Tarapacá
[karina.alarcon.castillo@gmail.com](mailto:karina.alarcon.castillo@gmail.com)

August 3, 2025
